# Female-age-dependent changes in the lipid fingerprint of the mammalian oocytes

**DOI:** 10.1093/humrep/deae225

**Published:** 2024-10-04

**Authors:** Simona Bisogno, Joanna Depciuch, Hafsa Gulzar, Maria Florencia Heber, Michał Kobiałka, Łukasz Gąsior, Adrianna Bereta, Anna Pieczara, Kinga Fic, Richard Musson, Gabriel Garcia Gamero, Maria Pardo Martinez, Alba Fornés Pérez, Martina Tatíčková, Zuzana Holubcova, Małgorzata Barańska, Grażyna Ewa Ptak

**Affiliations:** Malopolska Centre of Biotechnology, Jagiellonian University in Kraków, Kraków, Poland; Institute of Nuclear Physics, Polish Academy of Sciences, Kraków, Poland; Department of Biochemistry and Molecular Biology, Medical University of Lublin, Lublin, Poland; Malopolska Centre of Biotechnology, Jagiellonian University in Kraków, Kraków, Poland; Doctoral School of Exact and Natural Sciences, Jagiellonian University in Krakow, Kraków, Poland; Malopolska Centre of Biotechnology, Jagiellonian University in Kraków, Kraków, Poland; Malopolska Centre of Biotechnology, Jagiellonian University in Kraków, Kraków, Poland; Malopolska Centre of Biotechnology, Jagiellonian University in Kraków, Kraków, Poland; Malopolska Centre of Biotechnology, Jagiellonian University in Kraków, Kraków, Poland; Doctoral School of Exact and Natural Sciences, Jagiellonian University in Krakow, Kraków, Poland; Jagiellonian Centre for Experimental Therapeutics, Jagiellonian University in Krakow, Kraków, Poland; Malopolska Centre of Biotechnology, Jagiellonian University in Kraków, Kraków, Poland; Malopolska Centre of Biotechnology, Jagiellonian University in Kraków, Kraków, Poland; Doctoral School of Exact and Natural Sciences, Jagiellonian University in Krakow, Kraków, Poland; Malopolska Centre of Biotechnology, Jagiellonian University in Kraków, Kraków, Poland; Malopolska Centre of Biotechnology, Jagiellonian University in Kraków, Kraków, Poland; Malopolska Centre of Biotechnology, Jagiellonian University in Kraków, Kraków, Poland; Department of Histology and Embryology, Faculty of Medicine, Masaryk University, Brno, Czech Republic; Department of Histology and Embryology, Faculty of Medicine, Masaryk University, Brno, Czech Republic; Reprofit International, Brno, Czech Republic; Jagiellonian Centre for Experimental Therapeutics, Jagiellonian University in Krakow, Kraków, Poland; Faculty of Chemistry, Jagiellonian University in Kraków, Kraków, Poland; Malopolska Centre of Biotechnology, Jagiellonian University in Kraków, Kraków, Poland

**Keywords:** lipid droplets, oocyte, exosomes, carotenoids, *Gnpat*, lipid analysis, coherent anti-Stokes Raman spectroscopy

## Abstract

**STUDY QUESTION:**

Can oocyte functionality be assessed by observing changes in their intracytoplasmic lipid droplets (LDs) profiles?

**SUMMARY ANSWER:**

Lipid profile changes can reliably be detected in human oocytes; lipid changes are linked with maternal age and impaired developmental competence in a mouse model.

**WHAT IS KNOWN ALREADY:**

In all cellular components, lipid damage is the earliest manifestation of oxidative stress (OS), which leads to a cascade of negative consequences for organelles and DNA. Lipid damage is marked by the accumulation of LDs. We hypothesized that impaired oocyte functionality resulting from aging and associated OS could be assessed by changes in LDs profile, hereafter called lipid fingerprint (LF).

**STUDY DESIGN, SIZE, DURATION:**

To investigate if it is possible to detect differences in oocyte LF, we subjected human GV-stage oocytes to spectroscopic examinations. For this, a total of 48 oocytes derived from 26 young healthy women (under 33 years of age) with no history of infertility, enrolled in an oocyte donation program, were analyzed. Furthermore, 30 GV human oocytes from 12 women were analyzed by transmission electron microscopy (TEM). To evaluate the effect of oocytes’ lipid profile changes on embryo development, a total of 52 C57BL/6 wild-type mice and 125 *Gnpat^+/−^* mice were also used.

**PARTICIPANTS/MATERIALS, SETTING, METHODS:**

Human oocytes were assessed by label-free cell imaging via coherent anti-Stokes Raman spectroscopy (CARS). Further confirmation of LF changes was conducted using spontaneous Raman followed by Fourier transform infrared (FTIR) spectroscopies and TEM. Additionally, to evaluate whether LF changes are associated with developmental competence, mouse oocytes and blastocysts were evaluated using TEM and the lipid dyes BODIPY and Nile Red. Mouse embryonic exosomes were evaluated using flow cytometry, FTIR and FT-Raman spectroscopies.

**MAIN RESULTS AND THE ROLE OF CHANCE:**

Here we demonstrated progressive changes in the LF of oocytes associated with the woman’s age consisting of increased LDs size, area, and number. LF variations in oocytes were detectable also within individual donors. This finding makes LF assessment a promising tool to grade oocytes of the same patient, based on their quality. We next demonstrated age-associated changes in oocytes reflected by lipid peroxidation and composition changes; the accumulation of carotenoids; and alterations of structural properties of lipid bilayers. Finally, using a mouse model, we showed that LF changes in oocytes are negatively associated with the secretion of embryonic exosomes prior to implantation. Deficient exosome secretion disrupts communication between the embryo and the uterus and thus may explain recurrent implantation failures in advanced-age patients.

**LIMITATIONS, REASONS FOR CAUTION:**

Due to differences in lipid content between different species’ oocytes, the developmental impact of lipid oxidation and consequent LF changes may differ across mammalian oocytes.

**WIDER IMPLICATIONS OF THE FINDINGS:**

Our findings open the possibility to develop an innovative tool for oocyte assessment and highlight likely functional connections between oocyte LDs and embryonic exosome secretion. By recognizing the role of oocyte LF in shaping the embryo’s ability to implant, our original work points to future directions of research relevant to developmental biology and reproductive medicine.

**STUDY FUNDING/COMPETING INTEREST(S):**

This research was funded by National Science Centre of Poland, Grants: 2021/41/B/NZ3/03507 and 2019/35/B/NZ4/03547 (to G.E.P.); 2022/44/C/NZ4/00076 (to M.F.H.) and 2019/35/N/NZ3/03213 (to Ł.G.). M.F.H. is a National Agency for Academic Exchange (NAWA) fellow (GA ULM/2019/1/00097/U/00001). K.F. is a Diamond Grant fellow (Ministry of Education and Science GA 0175/DIA/2019/28). The open-access publication of this article was funded by the Priority Research Area BioS under the program “Excellence Initiative – Research University” at the Jagiellonian University in Krakow. The authors declare no competing interest.

**TRIAL REGISTRATION NUMBER:**

N/A.

## Introduction

Lipids in the form of lipid droplets (LDs) and membranes are present in all cellular compartments. Among the organic molecules that make up the cell—such as nucleic acids, proteins, carbohydrates, and lipids—lipids are the most prone to oxidation. Close interactions of lipids with proteins and DNA make lipid peroxidation potentially harmful to all cellular organelles. Because oxidative damage starts from lipids, with phospholipids being particularly sensitive, fragile membrane bilayers composed of phospholipids are first damaged. Beside plasmatic and nuclear membranes, membranous organelles and especially mitochondria are most prone to oxidation, in part because of their own contribution to ROS production (Olzmann and Carvalho, 2018). Relevantly, OS damage and mitochondrial dysfunction were noted in human oocytes (Ashwood-Smith and Edwards, 1996). A growing body of evidence implicates OS-mediated changes in mitochondria as a cause of the deterioration of oocyte quality (Pandey *et al.*, 2024). Damaged lipids can be transmitted from the oocyte to blastomeres during embryo cleavages, instilling fragility to newly formed cellular membranes and organelles, affecting lipid metabolism and signalling of the blastocyst. Membrane damage leads to the biogenesis of new components by LDs, where membrane building blocks are stored. Oxidative damage of lipids is marked by the accumulation of LDs (Liu *et al.*, 2015). As a storage compartment of membrane building blocks and a cytoplasmic hub for signaling (Barbosa *et al.*, 2015), LDs appear to be a major player in preimplantation development and embryo-maternal cross-talk. Here, we hypothesized that alterations to LF affect the developmental competence of the oocyte.

Conventional light microscopy assessments of human oocyte quality cannot detect OS, which represents a major problem in ART (Musson *et al.*, 2022). One setback for detecting OS damage in the oocyte is that modifications to mitochondrial and other bilayers is rarely accompanied by visual differences. Also LDs, being minimally represented in human oocytes, are usually overlooked in ART assessments. Thus, alterations to LDs and LF can be an important measure of functionality of the female gamete—the oocyte.

## Materials and methods

All chemicals, unless otherwise indicated, were obtained from Merck, Darmstadt, Germany.

### Human oocytes

A total of 48 germinal vesicle (GV)-stage oocytes derived from 26 women enrolled in a clinical egg donation program were used in the study. All donors were of Caucasian background and underwent screening against known hormonal and genetic factors that could negatively affect reproduction. Ovarian stimulation, oocyte retrieval, and denudation were performed as previously described (Trebichalská *et al.*, 2021). From all collected oocytes only those at the GV stage were used in our study (MII oocytes were used for clinical purposes). GV oocytes were washed in 0.4% (w/v) polyvinylpyrrolidone (PVP) in PBS and fixed in 4% paraformaldehyde for 20 min. Work was performed in compliance with institutional ethical approval no. 16/2016 and 1/2019 from Ethics Committee Faculty of Medicine Masaryk University. GV oocytes were provided for research following the informed consent of the donor.

### Animal studies

Animal experiments were performed on n = 25, 9–12 months old C57BL/6 mice for the Aged group, and n = 15, 3–4 months old C57BL/6 mice for the Control group; and on n = 125 *Gnpat* knockouts. For delipidation experiments, zygotes from n = 12, 3–4 months old C57BL/6×CBA hybrid mice were used because of their superior survival following manipulation compared with inbred C57BL/6 strain. Animals were maintained in temperature- and light-controlled conditions (22°C and 12 h light–dark cycle) and were provided with food and water *ad libitum*. The experiments were not randomized, and the investigators were not blinded to experiment allocation or outcome assessment. All experimental procedures were conducted according to the guidelines of European Community Regulation 86/609 and conformed to the Polish Governmental Act for Animal Care. Animal procedures were approved by II Local Ethical Commission of Kraków (permission no. 123/2018).

### Oocyte maturation

*Gnpat^−/−^* and *Gnpat^+/+^* females were euthanized, and cumulus-oocyte complexes were collected from the ovaries by needle puncturing in M2 media. Cumulus-oocyte complexes were matured in a media composed of MEM, 2 mM GlutaMAX, 0.3 mM sodium pyruvate, 5% foetal bovine serum, 1.5 IU/ml recombinant hCG and cultured in a humidified atmosphere of 5% CO_2_ in the air for 17 h.

### Parthenogenetic activation

The cumulus-oocyte complexes were mechanically denuded, and oocytes were placed in 400 μl of KSOMaa with 5 mM ionomycin for 5 min. Subsequently, the oocytes were washed three times in KSOM and then placed in KSOMaa with 2 mM 6-dimethylaminopurine (6-DMAP) for 3 h. The oocytes were then washed and placed in culture in 20 μl drops of KSOMaa covered with mineral oil and cultured in a humidified atmosphere of 5% CO_2_, 5% O_2_ in N_2_ for 72 h.

### Zygote delipidation

Zygotes were manipulated as previously described (Arena *et al.*, 2021). Briefly, 3–4 months old C57BL/6×CBA females were injected (i.p.) with 5 IU of pregnant mare serum gonadotropin (PMSG) followed 48 h later by an i.p. injection of 5 IU hCG to induce superovulation. Females were paired overnight with stud males. The zygotes were isolated from ampullae 23 h after hCG administration in M2 medium. Lipid polarization in the zygotes was obtained by centrifugation at 7000×*g* for 12 min at 37°C, with a preincubation of 10 min in modified M2 containing 1% bovine serum albumin and 10 μg/ml cytochalasin B. Centrifuged zygotes were allowed to recover for 1 h in KSOMaa and then manipulated. The zygotes were manipulated under a Leica inverted microscope equipped with Narishige micromanipulators (MO-108). The zygotes were manipulated based on a two-step protocol: the zona cut in KSOMaa and the removal of the lipids fraction or the same size fraction of cytoplasm in KSOMaa with 10 μg/ml cytochalasin B to produce delipidated (DEL) and non-delipidated (CTR) embryos, respectively. The resulting embryos were *in vitro* cultured in KSOMaa at 37°C under 7% O_2_ and 5% CO_2_ in N_2_ until day 4. A batch of day 4 embryos was destined for LD staining, another batch was transferred to pseudo-pregnant females, and the pregnancy rate was scored.

### Oocyte collection—AGED and CTR

Aged and control females were injected i.p. with 5 IU of PMSG followed 48 h later by i.p. injection of 5 IU hCG to induce superovulation. MII oocytes were isolated from ampullae 14 h after hCG administration in M2 medium and immediately fixed for analyses.

### Embryo collection—AGED and CTR

After pairing Aged and Control females with stud males overnight, vaginal plugs were scored the subsequent day. The plug-positive females were ethically sacrificed by cervical dislocation at day 4 *post coitum* to collect embryos. Embryos were collected by uterine flushing with M2 medium containing 0.4% (w/v) PVP (0.4% M2-PVP), 1 ml for each horn. A batch of the obtained embryos was fixed for further analyses, while another batch was transferred to pseudo-pregnant females (Arena *et al.*, 2021) and the pregnancy rate was scored.

### Embryo collection—Gnpat KO

After matings of 3–4 months old Gnpat^*−*^^/^^*−*^, Gnpat^+/^^*−*^, and Gnpat^+/+^ females by C57BL/6 stud males, the following day positive vaginal plugs were scored. Plug-positive females were destined for embryo retrieval at day 2 *post coitum*. Embryo collection was performed by oviduct dissection and uterine flushing with 0.4% M2-PVP, 1 ml for each horn.

### Embryo collection medium

At day 4 *post coitum*, the AGED and CTR embryos were collected by uterine flushing of pregnant females as described previously (Arena *et al.*, 2021). After embryo retrieval, the embryo collection media of each horn were centrifuged at 3500 *g* for 10 min at 4°C to remove cellular fraction and blood contamination. The supernatant was frozen at −80°C until exosome isolation and analysis.

### Isolation of exosomes

Exosomes were obtained from the supernatant of the embryo collection medium by differential centrifugation, according to published protocols (Witwer *et al.*, 2013). To remove debris, macroparticles, and apoptotic bodies the supernatant was thawed and centrifuged at 20 000 g for 30 min at 4°C. The resulting pellet, enriched in extracellular vesicles (EVs), was resuspended in 15 µl of PBS and stored at −80°C. Then the supernatant was transferred to 11 × 34 mm polycarbonate centrifuge tubes (Beckman Coulter Inc.) to perform the first ultra-centrifugation step at 100 000×g for 2 h at 4°C in an MLA-130 (Beckman Coulter, Inc.) rotor using an Optima MAX-XP ultracentrifuge (Beckman Coulter, Inc.); the supernatant was removed completely, and the obtained pellet was washed in 1 ml of PBS and centrifuged at 100 000 g for 1 h at 4°C. The final pellet enriched in small EVs (exosomes) was resuspended in 15 µl of PBS and stored at −80°C for flow cytometry, FTIR and FT-Raman analysis.

### Genotyping of Gnpat knock-out mice

Mice with targeted inactivation of *Gnpat* gene (MGI: 2670462, Gnpat^tm1Just^/Gnpat^tm1Just^) (Rodemer *et al.*, 2003), were kindly donated by Prof. Johannes Berger, Center for Brain Research, Medical University, Vienna, Austria. The strain was maintained on a C57BL/6×CD1 background. Experimental *Gnpat^−/−^* and *Gnpat^+/+^* littermates were obtained by mating of heterozygous animals. Mouse tail tissues collected from adult animals were used for genotyping. KAPA Mouse Genotyping kit (Sigma-Aldrich) was used for both the extraction of genomic DNA and PCR amplification, following the manufacturer’s instructions. The PCR reactions included three primers: OLI 1626: CGATACCTACTTTGTCCCAATTAGC; OLI 1627: GCTGGTCTCAAACAGCTACGTAGCTGA; OLI 1628: CGCATCGCCTTCTATCGCCTTCTTG. 100 ng of genomic DNA and 20 pmol of each oligo were added to KAPA genotyping mix (Sigma-Aldrich) in a final volume of 12.5 µl. After denaturation at 95°C for 3 min, PCR was performed using 35 cycles at 95°C for 15 s, 60°C for 20 s, 72°C for 50 s, followed by a final extension at 72°C for 7 min, resulting in a 700 bp product for the wt gene, and a 900 bp product for the *Gnpat^−/−^*; while heterozygous animals showed both 700 and 900 bp product. PCR products were analyzed by an electrophoretic run in 1.5% agarose gel.

### Coherent anti-Stokes Raman scattering microscopy

LDs distribution in oocytes was evaluated using coherent anti-Stokes Raman scattering (CARS) as described previously (Arena *et al.*, 2021). The multimodal nonlinear microscope used in this study consisted of a Leica DMi8 inverted microscope equipped with the Leica TCS SP8 CARS module and the Leica SP8 confocal module (Leica Microsystems). The Leica TCS SP8 CARS uses a tuneable pump laser with a tuning range of 780–940 nm, combined with a Stokes laser at 1064 nm, provided by Laser picoEmerald and integrated with a 750-mW power optical parametric oscillator.

#### Sample preparation

Oocytes in drops of 200 µl of PBS were placed in a Lab-Tek chamber (Thermo Scientific) for measurement. To detect LDs in the oocyte the optical parametric oscillator’s wavelength was tuned to 816.7 nm to serve as the pump beam, in combination with the 1064 nm Stokes beam to probe the CH_2_ stretch vibration. The signal was detected using a photomultiplier tube by collecting the photons in a forward direction through a 40× objective. Each sample was scanned across all dimensions in the *Z*-axis using a *Z*-step of 1.5 µm. All stacks from a single sample were processed to obtain a maximum projection image. The threshold was adjusted specifically for each sample to collect all positive signals. When necessary, LDs were separated manually in cases of obvious pixel overlay. Statistics regarding the LDs area and number were generated automatically by LAS X software (Leica Microsystems). The LAS X software was used to apply color-coded LD size masks to the maximum projection images for graphical observation of the LDs’ size distribution of the analyzed samples. The sample area was calculated using ImageJ (NIH) due to the limitations of the LAS X software.

### Confocal Raman imaging

Raman imaging was performed using WITec Confocal Raman Microscope (WITec alpha 300), operated via WITec Control Software. All spectra were collected with a laser excitation of 532 nm, a black-illuminated CCD camera, immersive objective (40X) (Nikon Fluor). All spectra were collected in the range 3670–0 cm^−1^, with a spectral resolution of 3 cm^−1^. The spatial resolution possible to achieve with this setup is approximately 0.36 μm. The size of the mapped area was 160 × 160 µm^2^. The sampling density for measured oocytes was 2 μm and the integration time was 0.1 s. Every sample was placed on a glass Petri dish in a 300- µl drop of PBS. Analysis of Raman imaging data was based on the integral intensity of selected bands to analyze the distribution of cellular components, i.e. organic matter (3015–2815 cm^−1^), saturated lipids (2850 cm^−1^), unsaturated lipids (3015 cm^−1^) and carotenoids (1157 and 1517 cm^−1^). The yellow color on Raman images corresponds to the highest relative intensity of the integrated band related to the distribution of a compound. Preprocessing of Raman spectra included Cosmic Ray Removal (CRR; filter size: 4; dynamic factor: 4) and Background Subtraction (BG; polynomial order: 3). The spectra prepared this way were analyzed by k-Means Cluster Analysis (KMCA) in the range of 1800–500 cm^−1^. K–Means cluster analysis was conducted with the Manhattan distance algorithm. Cluster analysis enabled spectra grouping into classes attributed to the major organelles in oocytes (i.e. cytoplasm, zona pellucida, peri-cytoplasmic region). The Raman spectra presented in the range of 3050–500 cm^−1^ were normalized using the Opus software (Bruker). For data presentation Origin 2021 (OriginLab Corporation) and GraphPad Prism 9 (GraphPad Software) were used.

### Fourier Transform infrared and FT-Raman spectroscopy

The Fourier transform infrared (FTIR) spectra were obtained using a Nicolet iS50 FTIR spectrometer (Thermo Scientific), using the attenuated total reflectance (ATR) technique. To obtain FTIR spectra, 4 µl of PBS solution containing a human or mouse oocyte or 4 µl of exosomes-rich PBS solution (see paragraph Exosome separation) was dropped into the CaF_2_ crystal. All samples were measured in the range of selected infrared radiation between 400 and 4000 cm^−1^ with the spectral resolution 2 cm^−1^ and 64 scans. The resulting spectra were normalized using a vector normalization by means of the OPUS 7.0 software.

FT-Raman spectra were measured by FT-Raman spectrometer Nicolet NXR 9650 equipped with a laser with 1064 nm wavelength. To obtain the spectra, a volume of 4 µl of each sample was dropped and left for 8 min to dry. Samples (oocytes or exosomes) were measured in the range from 150 to 3700 cm^−1^ with 4 cm^−1^ spectral resolution and 64 scans. The laser power was 1 W. Raman spectra were processed by the Omnic/Thermo Scientific software.

The average absorbance of infrared (IR) and Raman ranges between 2700 cm^−1^ and 3000 cm^−1^ and between 1500 cm^−1^ and 1700 cm^−1^ were calculated using Excel 2016 software. Data are presented as the mean ± standard error. For both spectroscopic techniques, spectra variation between the samples was analyzed using Principal Component Analysis (PCA). PCA analysis was calculated for the lipids region (2700 cm^−1^–3000 cm^−1^) as well as for the amide region (1300 cm^−1^–1650 cm^−1^) using the Past 3.0. software.

### Transmission electron microscopy

#### Human oocytes

Oocytes were fixed in 2.5% glutaraldehyde overnight at 4°C, washed in 0.1 M of cacodylate buffer, and treated in 1% OsO_4_ for 1 h. Samples were dehydrated through a graded series of ethanol, then infiltrated in pure resin (Poly/Bed 812, Polysciences, Inc., Warrington, USA) through growing concentrations of resin in: 100% propylene oxide; propylene oxide: resin 3:1 for 1 h; and then 1:1 overnight at room temperature. The samples were transferred into pure resin, and polymerization was carried out at 60°C for three days. Sections of ∼70 nm were cut by an ultramicrotome (Leica) contrasting with uranyl acetate and lead citrate. Images were captured by Tecnai Osiris 200 kV (FEI). LDs area was quantified using ImageJ segmentation tool (NIH).

#### Mouse oocytes

Oocytes were fixed in 2.5% glutaraldehyde in 0.1 M cacodylate buffer, then washed and subsequently post-fixed in 1% OsO_4_. For dehydration, oocytes were subjected to a graded ethanol series: beginning with 50% ethanol for 10 min; progressing to 70% for 10 min; 90% for another 10 min; followed by two changes in 96% ethanol for 10 min each; and ending with two 10-min immersions in 99.9% ethanol. After dehydration, oocytes were cleared in propylene oxide and infiltrated with an epoxy resin mixture before being embedded in pure resin. The polymerization of the resin blocks was carried out at 60°C for three days. Ultrathin sections with a thickness of approximately 70 nm were cut using an ultramicrotome, collected on copper grids, and double-stained with uranyl acetate and lead citrate for contrast enhancement. The prepared samples were examined and images were recorded using a JEOL JEM2100 HT transmission electron microscope.

### LD staining by BODIPY 493/503 and Nile Red

For LD staining, oocytes and blastocysts were fixed in 4% paraformaldehyde for 20 min and then incubated with 1 μg/ml BODIPY 493/503 (Invitrogen™) and 10 μg/ml Nile Red in 0.4% PBS-PVP, as previously described (Bisogno *et al.*, 2023). Mounted specimens were analyzed with a ZEISS LSM 880 Confocal Laser Scanning Microscope using a 20× Zeiss Plan-Apochromat Infinitive corrected objective with a numerical aperture of 0.8. Relative fluorescence signal intensities were analyzed using ImageJ and normalized to control.

### Flow cytometry

Annexin A5/FITC and CD63/PE Cyanine7 assays were performed using A50 Micro (Apogee) flow cytometer. Annexin A5/FITC (Beckman Coulter) was used to label the phosphatidylserine in the outer membrane leaflet of EVs. Two microlitres of previously prepared PBS solution enriched in exosomes (see section Isolation of exosomes) were incubated with Annexin A5/FITC and 100 µl of staining buffer for 15 min on ice. After incubation, another 100 µl of staining buffer was added. A dot plot cytogram with a green fluorescence signal for Annexin A5/FITC versus medium angle light scatter (MALS) was used to collect the data. A calibration bead mix (Apogee) containing beads of known sizes was used to scale the MALS signal first. Populations of Annexin A5-positive events were divided on the dot plot between three ranges: (i) <200 nm; (ii) 200–500 nm; and (iii) 500–650 nm. The absolute number of Annexin A5-positive events/µl for each size range was calculated by the cytometer’s software after the acquisition of 2.5–3 µl of sample.

## Results

### Woman-age-dependent LDs changes in oocytes

**Figure 1.**
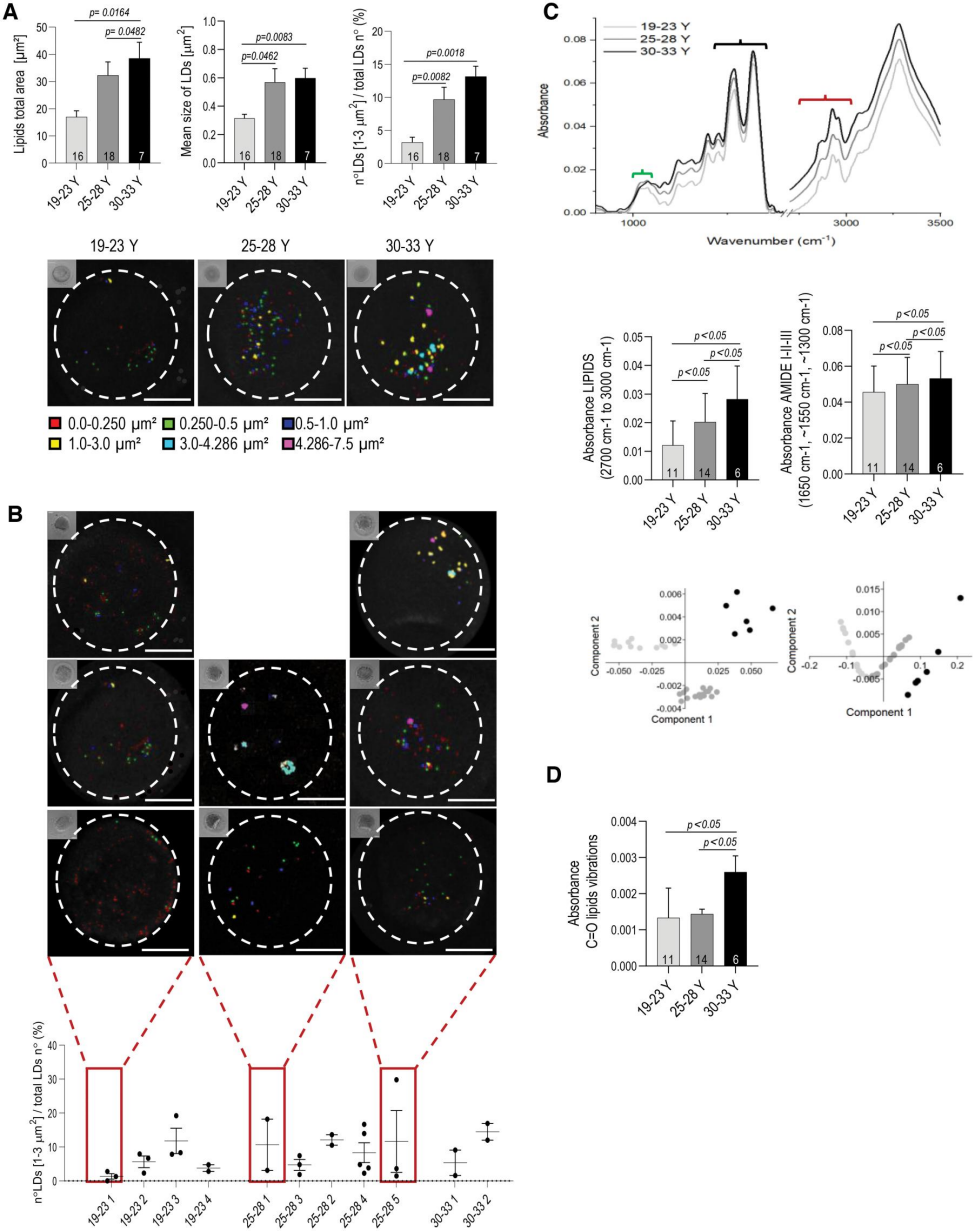
**Changes in oocyte LDs associated with woman’s ages.** (**A**) The histograms show the lipid fingerprint (LF) values of human oocytes, such as the total lipid area, mean lipid size and the number of LDs with dimensions between 1.0 and 3.0 μm^2^, increasing with woman’s age. The panel below shows representative coherent anti-Stokes Raman spectroscopy (CARS) images of oocytes of three women’s age groups: 19–23, 25–28, and 30–33 years old (Y). Scale bar 30 μm. n^o^ = number. The number inside the columns indicates the number of analyzed oocytes. Colors associated with each identified LDs size are indicated below. The corresponding CARS image oocyte in the bright field is shown in the upper left corner. Values represent mean ± SEM; one-way ANOVA, Kruskal–Wallis test. (**B**) Representative images and graphs indicating changes in LF in individual donors. Only donors with ≥2 oocytes were analyzed. Red squares indicate the coefficient of variation ≥1. (**C**) Lipid amount increases and phospholipid structure changes in oocytes as woman’s age advances: *Upper row*: mean FTIR spectra show absorbance of: phospholipids (∼1080 cm^−1^, ∼1250 cm^−1^)—green bracket; lipids functional groups (2700 and 3000 cm^−1^)—black bracket; and amide III (∼1300 cm^−1^) amide II (∼1550 cm^−1^), amide I vibrations (1650 cm^−1^)—red bracket. Phospholipid structural changes in oocytes (green bracket) from donors >23 years of age (dark grey line and black line) are indicated by the modification of peaks shape, while an increased amount of lipids (black bracket) is shown by increased absorbance (*y*-axis) in donors >23 years of age (dark grey line, and black line). *Middle row*: left graph shows FTIR absorbance related to lipid amount (2700–3000 cm^−1^); right graph shows FTIR absorbance related to Amide I-II-III amount (1650, ∼1550, ∼1300 cm^−1^). *Lower row*: corresponding principal component analysis (PCA) plots for FTIR range show clear distinction of three age ranges. (**D**) Average value of absorbance in FTIR range originating from C = O lipids vibrations indicative for lipid peroxidation. Numbers inside the columns indicate the number of analyzed oocytes collected from a total of 22 donors: 19–23 Y = 9 donors, 25–28 Y = 8 donors, 30–33 Y = 5 donors. mean ± SD; ANOVA, Kruskal–Wallis, Tukey’s *post hoc*.

Using Coherent anti-Stokes Raman scattering (CARS) microscopy, we identified woman-age-dependent progressive changes in the LF of GV stage oocytes represented by an increase in total LDs area, size, and number in three age groups: 19–23 versus 25–28 versus 30–33 years old (Fig. 1A and Supplementary movies 1, 2, and 3). CARS is a dye-free method which images lipids by revealing their characteristic vibrational contrast. The crucial advantage of this method is that it is fast and the oocyte remains unaffected. CARS can be used on living cells, a fact that constitutes its principal advantage in case of prospective use for clinics. However, due to the long distance between the center collecting the oocyte and our analysis (there are only a few CARS facilities in Europe) we could organize CARS analysis only following the fixation of oocytes. Nevertheless, we were able to reveal appreciable LF variations within individual donors (Fig. 1B, Supplementary movies 4, 5, 6, 7, 8, 9, 10, and 11). These demonstrable differences in oocyte LF within healthy women confirm the high sensitivity of CARS and give a strong basis for investigations of functional and dysfunctional LF by machine learning in the future.

To explore whether an altered LDs profile is associated with changes in lipids composition, we analyzed FTIR spectra of human oocytes from three age ranges (Fig. 1C and D). FTIR analysis is a cell-destructive biochemical technique that uses infrared light to scan the oocyte and observe its chemical properties. This analysis demonstrated the woman-age-dependent increase in levels of phospholipids and amides, both of which are constituents of membrane bilayers (Fig. 1C). In somatic cells, changes in phospholipids and amides occur as a consequence of OS and aging (Gaveglio *et al.*, 2016; Beharier *et al.*, 2020; Wang *et al.*, 2022). The oxidation of an amine leads to the construction of stable amide bonds (Nagaraaj and Vijayakumar, 2019). For example, OS-driven changes in the level of phosphatidylethanolamine induce anandamide synthesis (Beharier *et al.*, 2020). High concentrations of anandamide are associated with implantation failure, trophoblast dysfunction, and embryo growth arrest (Maccarrone *et al.*, 2002; Abbas *et al.*, 2020; Beharier *et al.*, 2020). In order to prove that woman-age-dependent LF changes in oocytes are a consequence of OS, we analyzed absorbance values in FTIR ranges originating from C = O lipids vibrations, indicative of lipid peroxidation (Fig. 1D). This analysis demonstrated an increased level of peroxidized lipids in oocytes from older women (≥30 years old).

**Figure 2.**
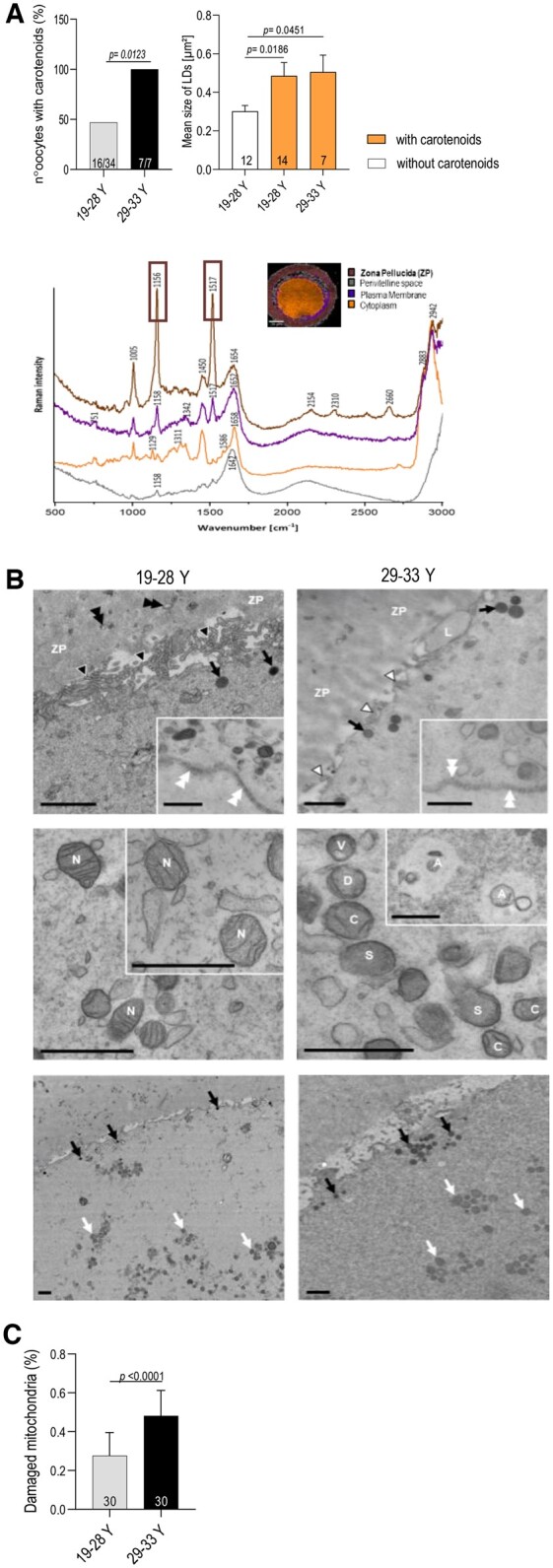
**Demonstration of age-dependent composition changes in human oocytes.** (**A**) Left histogram shows that carotenoids presence in ZP is associated with more advanced women’s age (Fisher test, *P* = 0.0123). Numbers inside the columns indicate oocytes with carotenoids out of total oocytes analyzed. The right graph shows an increase in mean LDs size (analyzed by CARS) in oocytes with carotenoids from both younger and older women. Number of oocytes analyzed is noted inside the column. ANOVA, Kruskal–Wallis. *Below the graphs*: Representative KMC analysis and average Raman spectra in the range of 3050–500 cm^−1^ of human oocytes (29–33 Y) with carotenoids showing the different oocytes parts (zona pellucida, perivitelline space, plasma membrane, and cytoplasm) with related chemical compounds peaks. Integration Raman band 1156 and 1517 cm^−1^ (indicated in squares) in ZP is indicative for carotenoids. (**B**) Representative electron micrographs of human oocytes demonstrating structural alteration of plasmatic membranes such as lower and structural alteration of microvilli of the plasma membrane from a long and thin (black arrowheads) to a short and bulbous appearance (white arrowheads), loss of membrane integrity and extraction of cytoplasmic lobes (L), thicker, undulated and compact nuclear membrane (white double arrowheads) in oocytes from younger donors. The zona pellucida (ZP) is less electron dense in older donors. *Middle row:* Morphological changes in mitochondria: normal morphology (N) in oocytes from younger donors and mitochondrial swelling (S), decreased electron density within the matrix (D), vacuolization (V), cristae alterations (the cristae are thinner and misoriented and/or decreased in number) (C) and degradation of mitochondria inside autophagosomes (A). *Lower row:* accumulation of LDs (black arrows) and swollen mitochondria (white arrows) in oocytes from older donors; number of fields counted >130. Scale bars = 1 µm. (**C**) Histogram shows higher presence of morphologically changed mitochondria in oocytes from older donors analyzed by TEM; number of fields counted >80/group; Student’s *t*-test.

The demonstration of an increased level of peroxidized lipids in oocytes from older women led us to search for the marker of lipid peroxidation using non-destructive cell imaging. Because lipid peroxidation disrupts cellular retinoic acid (RA) homeostasis (Lee *et al.*, 2008) and because RA insufficiency is related to aging (Gaveglio *et al.*, 2016), we hypothesized that retinoid signal detection may be useful for evaluation of OS in oocytes. Using non-invasive label-free confocal Raman imaging we were able to detect carotenoids (retinol precursors) in zona pellucida (ZP) of oocytes from all the older donors (29*–*33 years old) and in part of ZP of oocytes from the younger donors (Fig. 2A, left graph). The presence of carotenoids in ZP was associated with increased LDs mean size in oocytes from both the younger (19–28 years old) and older (29–33 years old) donors (Fig. 2A, right graph). There were no carotenoids in ZP of oocytes from the younger donors (19–28 years old) with a low LDs mean size. Our findings therefore indicate that increased LDs size may be associated with carotenoid mobilization, triggered by OS in the oocyte. RA deficiency in the oocyte, caused by lipid peroxidation and evidenced by an increased LDs size, can only be corrected by an external supply of RA precursors. Because RA is essential for meiotic resumption (Wang *et al.*, 2023) and zygotic progression (Iturbide *et al.*, 2021), carotenoid accumulation in ZP may be an attempt to restore oocyte functionality. Our successful detection of carotenoids—modulators of OS—makes oocyte assessment by Raman imaging a reliable tool to distinguish the quality of oocytes, especially if combined with their evaluation by CARS.

In order to reveal OS-dependent structural changes in the oocyte membranes, we next characterized human oocytes using TEM (Fig. 2B). We noted several alterations of the plasma membrane including a decreased size and number of microvilli in the oocytes of older donors. We also observed changes in the thickness and undulation of the nuclear membrane, suggesting OS-driven damage of the bilayers (Fig. 2B, panel upper row). It is known that damage to the lipid bilayers and the accumulation of carotenoids—which rigidify membranes—can cause cellular organelle alterations and affect nuclear homeostasis (Gabrielska and Gruszecki, 1996). The rigidity of human oocyte membranes has previously been shown to be associated with the misexpression of genes important for chromatid segregation and the spindle assembly checkpoint (Yanez *et al.*, 2016). From all cellular membranes evaluated, the most apparent damage we observed concerned mitochondrial membrane defects in oocytes from women ≥30 years old (Fig. 2B, panel middle row and Fig. 2C). Mitochondrial damage is considered the major determinant of oocyte aging (May-Panloup *et al.*, 2016). Age-related mitochondrial profile alteration has previously been reported, however, that previous investigation was conducted on oocytes from women at a more advanced age (Muller-Hocker *et al.*, 1996). The origin of observed mitochondrial swelling and cristae alterations is linked to the oxidative changes of phosphatidylethanolamine located on the inner membrane of the mitochondria (Beharier *et al.*, 2020). Furthermore, the altered electron density in the ZP of oocytes from older women (Fig. 2B, panel lower row) indicates a change in their physical properties. These observed structural changes in the ZP of oocytes from older donors—further confirmed by the presence of carotenoids—could explain the age-dependent rigidity of ZP (Yanez *et al.*, 2016). ZP changes negatively affect the success of human embryo development in terms of fertilization and hatching (Yanez *et al.*, 2016).

### Low developmental competence of mouse oocytes with altered LF

**Figure 3.**
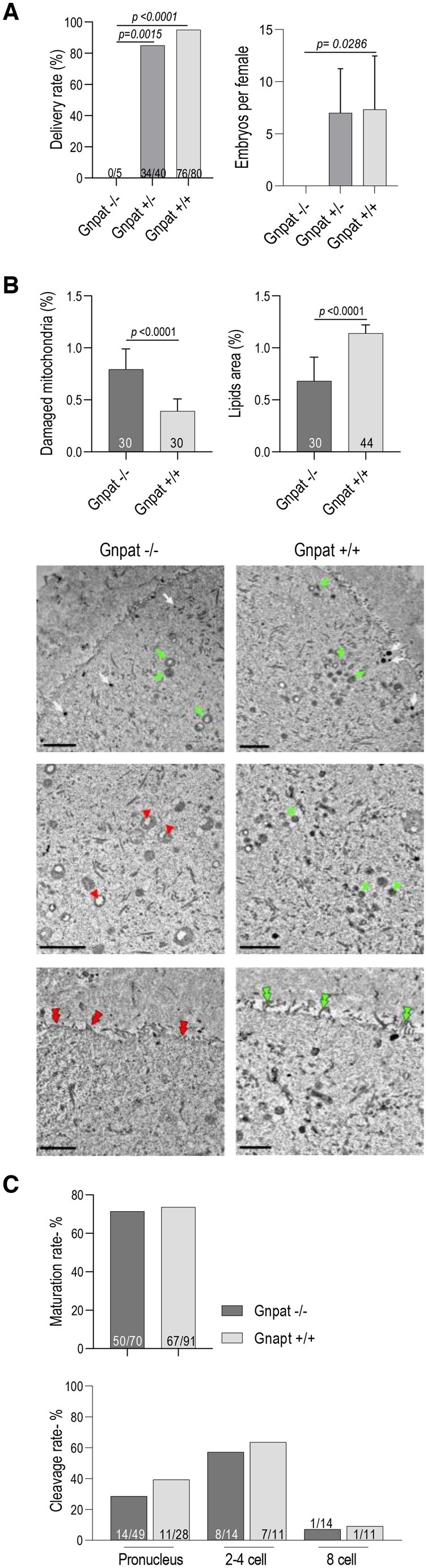
**Low developmental competence of mouse oocytes with altered LF.** (**A**) *Left graph:* Histogram shows pregnancies with deliveries rates of *Gnpat^−/−^, Gnpat^+/−^*, and *Gnpat^+/+^* mice. Numbers inside the columns indicate n of females which delivered offspring out of n of females mated; Chi-square test. *Right graph:* Rate of embryos flushed from the uteri of mice at 2 days *post coitum*; mean ± SEM; ANOVA, Kruskal–Wallis test. (**B**) Left histogram shows higher number of morphologically changed mitochondria in oocytes from *Gnpat^−/−^* analysed by TEM. Right histogram shows lower number of LDs in oocytes from *Gnpat^−/−^* (Mann–Whitney–Wilcoxon test). *Panel below*: representative electron micrographs demonstrating: (*first row*) reduced size and number of LDs (white arrows) and swollen mitochondria (green arrows) in oocytes from *Gnpat^−/−^* mice; (*second row*) mitochondria (indicated by green arrowheads in *Gnpat^+/+^*) are characterized by morphological changes and vacuolization (red arrowheads) in *Gnpat^−/−^* oocytes; (*third row*) morphological defects in plasmatic membranes, the lower number and length of microvilli (red double arrowheads) of the plasma membrane in comparison to normal microvilli (green double arrowheads). Number of fields counted >35/group. Scale bars = 2 µm. (**C**) Upper graph shows the proportion of GV oocytes collected from *Gnpat^−/−^* and *Gnpat^+/+^* ovaries which progressed to MII stage following *in vitro* maturation. No difference between two groups—Fisher-test. Number inside the columns indicate n of MII oocytes following 18 h of maturation. Lower graph shows similar rate of development of *Gnpat^−/−^* and *Gnpat^+/+^* oocytes up to 8 cell-stage, following parthenogenetic activation *in vitro* (Chi-square test).

We next sought to determine how qualitative changes in the LF shape oocyte competence. To do this, we used a mouse knock-out (KO) model deficient for ether lipids, which constitute around 20% of phospholipids in mammals. These KO mice lack a key enzyme in ether lipid biosynthesis—Glycerone-phosphate O-acyltransferase (Gnpat) (Rodemer *et al.*, 2003). *Gnpat^−^*^/^^*−*^ males are known to be infertile, while observations regarding fertility of females are limited (Rodemer *et al.*, 2003). From the breeding of eight pairs of *Gnpat^+/−^* male and female mice all offspring which survived to adulthood (n = 125) were subjected to genotyping. Their genotyping revealed only 5 *Gnpat^−/−^* females, equaling 4% of all genotyped mice. None of the *Gnpat^−/−^* females delivered following repeated breeding attempts (Fig. 3A, left graph). In order to verify if this pregnancy failure is a consequence of an inability of embryos to implant, or of other embryo problems, uteri of *Gnpat^−/−^* females were flushed at 2 days *post coitum*. No embryos were present (Fig. 3A, right graph). We next collected GV stage oocytes from *Gnpat^−/−^* and wild type females and subjected them to TEM analysis (Fig. 3B). TEM analysis demonstrated that phospholipid compositional changes (i.e. lack of ether lipids) in *Gnpat^−/−^* oocytes are a causative factor for decreased LDs mean size and changes of electron density. *Gnpat^−/−^* oocytes were also characterized by structural defects in plasmatic membranes and mitochondria (Fig. 3B). Overall, LF changes were similar to those observed in oocytes from older women (Fig 2B). We next aimed to investigate the developmental competence of remaining *Gnpat^−/−^* oocytes via maturation and activation of oocytes *in vitro* (Fig. 3C). We found that *Gnpat^−/−^* oocytes (at GV stage) were able to mature to metaphase II stage and to progress through 2–3 cleavages following parthenogenetic activation (Ptak *et al.*, 2014). Still, it is not known if *Gnpat^−/−^* oocytes are able to be fertilized. Considering that ether lipids are relevant for membrane fusion (Dean and Lodhi, 2018), we believe the process of fusion between oocyte and sperm may be compromised in *Gnpat^−/−^.*

**Figure 4.**
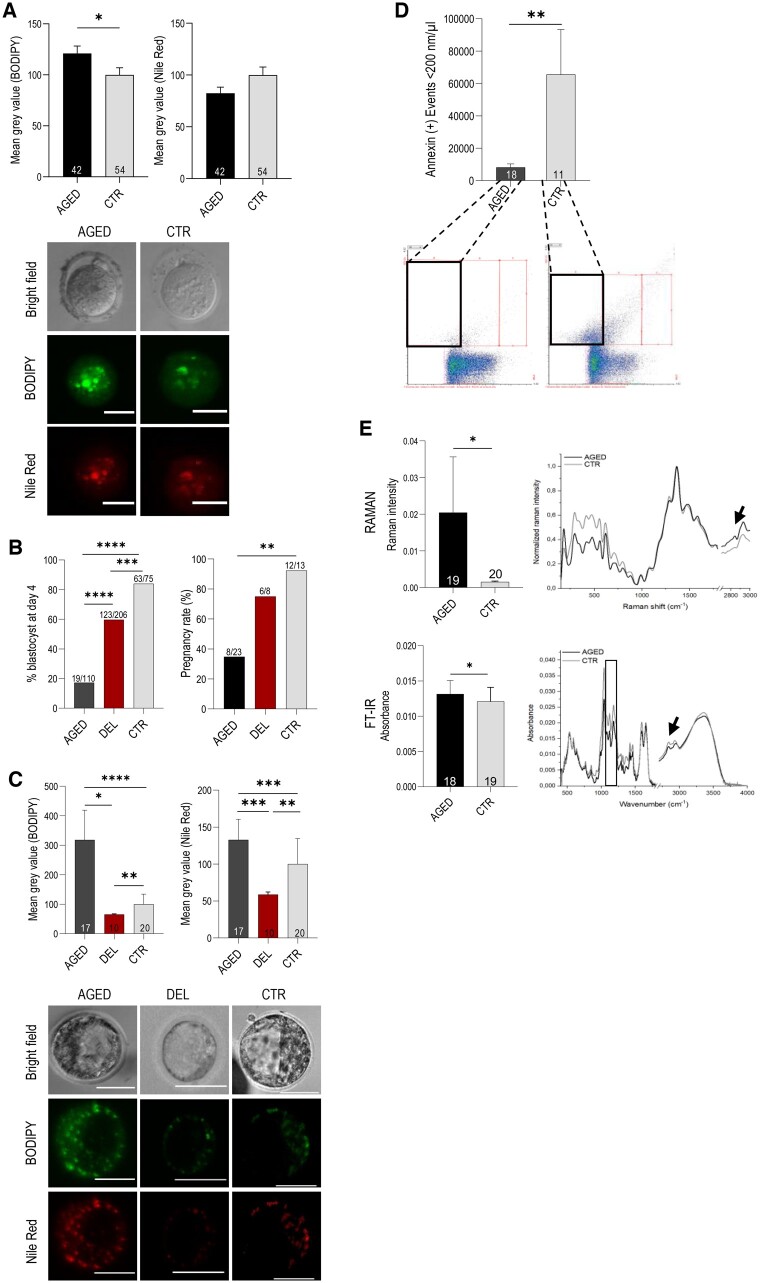
**An altered LF is maintained in mouse blastocysts and exosomes.** (**A**) Histograms and representative confocal images show increased lipid level in eggs (MII oocytes) collected from AGED and young (CTR) mice subjected to superovulation. Values represent mean ± SEM, Mann–Whitney test, *P < 0.05. Numbers inside the columns indicate number of oocytes. The panel shows representative images of oocytes stained with BODIPY and Nile Red. Scale bar 50 µm. (**B**) *Left graph:* The histogram shows the proportion of blastocysts obtained from aged females (AGED), young females (CTR) and following delipidation of zygotes obtained from young females (DEL). Chi-square test. *Right graph:* The histogram shows pregnancy of AGED, DEL, and CTR groups. Mean ± SEM; 1-way ANOVA, two-tailed unpaired Student’s *t*-test, **P < 0.01, ***P < 0.001, ****P < 0.0001. Numbers above the column indicate number of pregnant females. Histograms show increased and decreased lipid levels in blastocysts from AGED, DEL, and CTR groups stained by BODIPY and Nile Red. Values represent mean ± SEM, ANOVA, Kruskal–Wallis, *P < 0.05, **P < 0.01, ***P < 0.001, ****P < 0.0001. Numbers inside the columns indicate number of blastocysts. The panel shows representative images of stained blastocysts from AGED, DEL, and CTR groups. Scale bar 50 µm. (**C)** The histogram and representative cytograms (flow cytometry) demonstrating decreased concentration of annexin-positive particles <200 nm (annexin marks exosomes, black square) isolated from the embryo collection media of preimplantation blastocysts from AGED mice (<200 nm; annexin marks exosomes). Number of samples is indicated inside the column; mean ± SEM; 1-way ANOVA, two-tailed unpaired Student’s *t*-test. (**D)** *Upper row*: the histogram and Raman spectra show an increased amount of lipids in exosomes from AGED group. Black arrow indicates higher mean value of Raman intensity corresponding to CH_2_ and CH_3_ lipids vibrations (range between 2700 and 3000 cm^−1^). Mean ± SD; ANOVA, Kruskal–Wallis, Tukey’s post hoc. *Lower row*: The histogram shows higher absorbance of lipids that confirm higher lipid level in exosomes from AGED group. Additionally, the spectra show mean value of FTIR absorbance originating from lipid vibrations range between 2700 and 3000 cm^−1^ (black arrow), phospholipids (1080 cm^−1^) (evidenced in square) and amides (1250, 1500, 1750 cm^−1^) in AGED group. Modification of the composition of lipids in exosomes form AGED group are evidenced by changes in the peaks position corresponding to lipids (2700–3000 cm^−1^) (black arrow). Mean ± SD; ANOVA, Kruskal–Wallis, Tukey’s post hoc, *P < 0.05.

We next checked how quantitative changes of LF influence embryo development. To do this, we used two models of oocytes: with the increased and decreased lipid content represented by non-fertilized MII oocytes from aged mice and delipidated zygotes from young mice, respectively. MII oocytes and zygotes were collected from the mice subjected to superovulation. Consistent with our previous indication (Musson *et al.*, 2022), here we confirm that altered LF is also present in MII oocytes derived from aged mice (Fig. 4A). Zygotes obtained from young mice were mechanically delipidated following centrifugation, which allowed the removal of at least 60% of total LDs (Bisogno *et al.*, 2020; Arena *et al.*, 2021). The developmental potential of lipid-deficient embryos was not affected either before implantation or after their transfer to synchronized recipients (mouse: Fig. 4B; pig (Nagashima *et al.*, 1995)). However, our previous study showed that embryo delipidation causes pregnancy failure in mice when the implantation is experimentally delayed for at least 5 days (Arena *et al.*, 2021). This suggests that lipid-deficient embryos may have insufficient energy resources to implant, or that lipid-mediated embryonic signaling is insufficient. Considering the poor development of *Gnpat^−/−^* embryos *in vitro* (Fig. 3A) and more successful development of delipidated embryos (Fig. 4B), it seems that qualitative changes to LF in *Gnpat^−/−^* mice have more serious consequences for the embryo than quantitative changes of lipids following delipidation. Taken together, these results indicate that lipid compositional changes lead to the developmental failure of embryos, while a decreased lipid level is less likely to compromise embryo development. As we demonstrated, the oocytes from older women represent a mixed phenotype: both quantity and composition of lipids are altered (Figs 1 and 2). Quantitative and qualitative alterations of LF are linked with reduced development of mice embryos from aged females (Fig. 4B).

### Altered LF is maintained until the blastocyst stage

We next aimed to examine whether altered LF persists throughout the preimplantation development or is corrected to a normal level during embryo development (Fig. 4C). We found that both the increased and decreased lipid levels (in oocytes originating from aged mice and in delipidated zygotes, respectively) were maintained at the blastocyst stage. LF changes can therefore persist throughout early development and may have long-term health consequences. In the absence of a (maternal) supply of new building blocks for de novo lipogenesis, any embryonic damage cannot be repaired. This would explain why the likelihood of aberrations in the earliest-developing cell populations—such as the central nervous system and the heart—is higher than in the organs which develop later (Orenzo *et al.*, 1999; Mitchell *et al.*, 2004; Udine *et al.*, 2021), once new undamaged maternal (lipid) resources are delivered to the embryo through the placental bloodstream.

### Defective exosome secretion prior to the implantation of embryos from aged females

Exosomes are lipid bilayer-enclosed nano-sized vesicles produced by various types of cells. Embryonic exosomes are responsible for preimplantation signaling with the uterus (Desrochers *et al.*, 2016). We sought to determine if the secretion of exosomes prior to implantation is altered in aged mice. To do this, embryos were flushed from the uteri of aged (9–12 months old) females at 4 days *post coitum* (dpc). Exosomes isolated from this embryo collection media were subjected to quantitative evaluations using flow cytometry (Fig. 4D). Annexin was used to label the phosphatidylserine in the outer membrane leaflet of exosomes and the annexin-positive events/µl were calculated. Evaluations revealed a low level of secretion of preimplantation exosomes obtained from the uteri of aged mice. Subsequently, we used two vibrational spectroscopy techniques: FT-Raman and FTIR spectroscopy—techniques that can measure the vibrational energy of the bonds in the sample to detect structural changes in the exosomes. We demonstrated a higher level of phospholipids and amides in the exosomes of uteri from aged females prior to the embryo implantation (Fig. 4E). As mentioned in the first part of our study, an increased level of amides such as anandamide is associated with implantation failure, trophoblast dysfunction, and early miscarriage (Maccarrone *et al.*, 2002; Beharier *et al.*, 2020). This may explain recurrent implantation failure of females at advanced age (Fig. 4B). Our results suggest that altered LF is associated with disrupted embryonic exosome secretion and impaired signaling of embryo presence in the uterus, which thus may compromise the implantation. This finding contributes to the current debate on what is the critical factor causing implantation failure in women of advanced age, suggesting a rule of insufficient embryo quality and signaling rather than impaired endometrial function (Pathare *et al.*, 2023).

## Discussion

It is probably human nature to label as ‘junk’ the elements of a system the function of which is yet unknown. ‘Junk DNA’ comes to mind, once considered non-coding sequences, despite the fact that they make up 98% of cellular DNA (Ohno, 1972). The first observations of LDs in mammalian oocytes led to similar conclusions as the situation with the non-coding DNA, as LDs were suggested to be evolutionary-maintained remnants lacking a critical function. Furthermore, the non-essential nature of LDs for embryo development was convincingly demonstrated by their removal from pig zygotes without affecting developmental competence (Nagashima *et al.*, 1995). Indeed, the mammalian embryo initially uses carbohydrates rather than lipids for metabolism. The transition from carbohydrate to lipid metabolism occurs at the blastocyst stage, concomitantly with the production of exosomes (Arena *et al.*, 2021). In addition, 50 years ago exosomes themselves—similar to non-coding DNA and LDs—were assumed unnecessary, mistakenly considered as damaged debris removed by the cell (Schrier *et al.*, 1971). Nowadays, LD’s regulation of embryonic exosome secretion remains mysterious. We recently demonstrated the temporal coordination of processes involved in the LDs mobilization and exosome biogenesis by mouse blastocysts at the start of preimplantation signaling (Arena *et al.*, 2021). Here we demonstrate the link between the defective oocyte LDs profile and deficient exosome secretion prior to the implantation of embryos in aged females. The relation between LDs and exosomes can be best described by picturing an embryonic invasion of the uterine wall, similar to a tumor invasion: both embryo implantation and tumor invasion are preceded by the LDs accumulation (Cruz *et al.*, 2020) and followed by a high secretion of exosomes (Desrochers *et al.*, 2016; Record *et al.*, 2018; Watanabe *et al.*, 2010). Our study suggests that oocyte LF-alteration-driven deficiency in the exosome secretion will negatively affect embryo implantation. In this view, our study sheds new light on the relevance of the LF of the original cell—the oocyte—in shaping the functionality of the embryo.

Although studies on the preimplantation embryonic signaling via exosomes are limited, there are some reports showing that increasing the number of embryos—therefore increasing exosome secretions—improves implantation success rates. For example, it is well recognized in ART that the transfer of two human embryos to the uterus instead of one ensures a higher pregnancy rate (Adamson and Norman, 2020). However, any further increase of the number of transferred embryos does not result in an additional increase in the pregnancy rate and remains similar to the pregnancies resulting from the transfer of two embryos (Milki *et al.*, 1999)^.^ This suggests that when the level of embryonic secretions is sufficient to signal embryo presence in the uterus, an additional (third) embryo is superfluous for maternal recognition of pregnancy. Moreover, in pigs, it has long been demonstrated that at least four embryos present in the uterus is the minimum signal for implantation to occur (Lambert *et al.*, 1991). With fewer embryos, implantation will not start. It is plausible therefore, that uterine recognition of the embryo can be compromised in the event of deficient exosome secretion, e.g. following the transfer of low-quality embryos from an older female. Further studies on the regulation of the secretion and composition of embryonic exosomes and their role in maternal recognition of pregnancy are needed.

So far, LF variation and its consequences on human oocyte competence has not been studied. Recent studies on animal models show the utility of Raman spectroscopies for the evaluation of oocyte lipid profiles (Bogliolo *et al.*, 2012; Davidson *et al.*, 2013; Jimenez *et al.*, 2022). Relevantly, a number of studies have demonstrated the use of Raman spectroscopy to verify oxidative damage and age-related changes in mouse oocytes (Bogliolo *et al.*, 2013; Ishigaki *et al.*, 2021). Altogether, these demonstrable woman-age-dependent changes in oocyte LF are characterized by both qualitative and quantitative modifications in the lipids and structural defects in the membranes and membranous organelles, such as the mitochondria.

The consequences of the OS-driven lipid damage to various cellular compartments require elucidation, especially in relation to biological membranes and possible links to DNA modifications. As a major component of membranes, phospholipids are the most prone to oxidative damage. Oxidized lipids diffuse over large distances in membranes and alter their properties, leading to the loss of mitochondrial integrity and the accumulation of lipid peroxidation products (LPO) (May-Panloup *et al.*, 2016; Beharier *et al.*, 2020). OS-driven damage to lipid membranes and accumulation of LPO in the cytoplasm as a consequence of OS may render inefficient the recently-developed strategies of mitochondrial addition. This rejuvenating therapeutic treatment has been developed to rescue oocytes by transferring healthy mitochondria into them, in order to increase their functionality and prevent mitochondrial disease (Minczuk *et al.*, 2008). However, if mitochondria are damaged as a consequence of the OS condition in the oocyte (Smits *et al.*, 2023), then such conditions will inevitably lead to similar damage of freshly injected mitochondria, caused by the high level of LPO in the cytoplasm. In such cases, germline nuclear transfer to the ooplasm of young donors instead of mitochondrial transfer may be more recommended to rescue poor embryo development associated with advanced maternal age (Tang *et al.*, 2020). Mitochondrial membrane dysfunction is strongly associated with OS, and together with the accumulation of mtDNA mutations, plays a key role in the deterioration of oocyte functionality (May-Panloup *et al.*, 2016; Beharier *et al.*, 2020). LPO reacts with DNA, causing the formation of DNA adducts and chromosome loss and/or duplication (Williams *et al.*, 2006; Zarkovic *et al.*, 2013). LPO is accumulated both in oocytes from aged mice (Mihalas *et al.*, 2018) and in so-called ‘Down syndrome mice’ (Ishihara *et al.*, 2009). Advanced maternal age at conception is a major risk factor for Down syndrome (Antonarakis *et al.*, 2020) and the pro-mutagenic action of LPO is involved in aneuploidy occurrence (Ishihara *et al.*, 2009). Considering the role of lipid membranes in supporting genome stability (Otsuka *et al.*, 2016; Hwang *et al.*, 2019; Kinugasa *et al.*, 2019), oxidative damage may lead to deficiencies in genome shielding. In fact, aneuploidies occur more frequently in the most exposed parts of chromosomes: those which are located near the nuclear membrane (McCoy *et al.*, 2015). A direct relationship between nuclear chromosome peripheral position and segregation error frequencies was recently noted (Klaasen *et al.*, 2022).

Another consequence of lipid peroxidation is inadequate histone assembly. Histone acetylation and deacetylation require cofactors such as lipid-derived acetyl-CoA (Shvedunova and Akhtar, 2022). Inadequate histone deacetylation causes aneuploidy in mice oocytes (Akiyama *et al.*, 2006; Legoff *et al.*, 2021). Altogether, our study indicates oxidative damage of lipids in oocytes from older females as one possible explanation of oocyte aneuploidy.

Studies on lipids in human oocytes are limited, likely because its LD content, being the lowest among mammals, is difficult to track. Recent advances in non-invasive chemical imaging and developments of laser sources have made possible real-time examination of oocytes based on vibrational spectroscopy. Emerging applications, such as (proposed here) non-invasive analysis LF assessment, raise many exciting possibilities for biology and medicine. Our original study demonstrated that LF in the human oocyte can now be evaluated using non-destructive cell imaging. The reliability of LF analysis was here confirmed by biochemical and TEM methods. Due to the unavailability of MII human oocytes, this study was performed using the GV stage, which limits our observation to immature female gametes. However, here using a mouse model we were able to confirm consistent changes in mouse MII oocytes and blastocysts. Our finding opens the potential for new diagnostic measures by establishing live-cell quality assessment of oocytes based on LF evaluation. The elaboration of software able to recognize and grade oocytes based on LF may turn out to be the most sensitive indicator of oocyte functionality. Lastly, our investigation sheds new light on a possible cause-and-effect relationship between LDs and exosomes in shaping the implantation ability of the embryo. These findings will likely ignite further studies in developmental biology and reproductive medicine fields.

## Supplementary Material

deae225_Supplementary_movie_1

deae225_Supplementary_movie_2

deae225_Supplementary_movie_3

deae225_Supplementary_movie_4

deae225_Supplementary_movie_5

deae225_Supplementary_movie_6

deae225_Supplementary_movie_7

deae225_Supplementary_movie_8

deae225_Supplementary_movie_9

deae225_Supplementary_movie_10

deae225_Supplementary_movie_11

## Data Availability

The data underlying this article are available in the article and in its online supplementary material.
